# Association between triglyceride-glucose (TyG) index and risk of depression in middle-aged and elderly Chinese adults: Evidence from a large national cohort study

**DOI:** 10.17305/bb.2024.11800

**Published:** 2025-01-15

**Authors:** Zhang-Yang Xu, Hong Zheng, Zi-Jun Pan, Shou-Yi Hu, Yun-Xia Wang, Wen-Jun Su

**Affiliations:** 1 Department of Stress Medicine, Faculty of Psychology, Second Military Medical University, Shanghai, China; 2 Department of Nautical Psychology, Faculty of Psychology, Second Military Medical University, Shanghai, China; 3 Department of Medical Psychology, Changzheng Hospital, Naval Medical University, Shanghai, China; 4 Department of Endocrinology, Chinese PLA General Hospital, Medical School of Chinese PLA, Beijing, China

**Keywords:** Depression, triglyceride-glucose index, TyG index, cox regression model, longitudinal study, biomarker, endocrine

## Abstract

Insulin resistance (IR) has been proposed as a contributing factor to major depressive disorder (MDD), with previous studies reporting a positive correlation between triglyceride-glucose (TyG) a proxy indicator of IR and MDD. However, limited information is available regarding their longitudinal association. This study aimed to clarify the connection between TyG levels and depression risk, as well as explore its predictive potential. A total of 3021 participants without a prior history of depression were recruited from the China Health and Retirement Longitudinal Study and followed for seven years. Participants were categorized into tertiles based on their TyG levels. The cumulative hazard of depression was analyzed using Kaplan–Meier curves, while cox regression analyses and multivariable-adjusted restricted cubic spline (RCS) curves were employed to assess the relationship between TyG levels and depression risk. Stratified analyses across various subgroups were also conducted to confirm the robustness of the conclusions. Over the follow-up period, 1782 participants (58.9%) developed depression, with incidence rates of 30.2%, 34.0%, and 35.8% in tertiles 1, 2, and 3, respectively. After adjusting for confounding factors, each 1-unit increase in TyG was associated with a significantly higher risk of depression. RCS curve analysis revealed a compelling dose-response relationship between TyG levels and depression susceptibility. These findings indicate that elevated TyG levels are strongly associated with an increased risk of depression and could serve as a reliable biomarker for assessing depression risk. These insights provide valuable guidance for developing more effective strategies for the prevention and treatment of depressive disorders.

## Introduction

Depression is a prevalent mental health issue characterized by persistent feelings of sadness, loss of interest, and diminished motivation. In severe cases, it can lead to self-harm or suicidal behavior [1]. The World Health Organization has identified depression as the leading contributor to the global burden of disease, affecting a staggering 280 million people worldwide. Epidemiological data reveals that the lifetime prevalence rate of depression in China’s population is approximately 3.4% [2]. Emerging evidence strongly suggests that metabolic dysregulation plays a significant role in the onset of depression. Additionally, mounting clinical research highlights a bidirectional relationship between metabolic syndrome (MetS) and depression. Elevated levels of glucose and triglycerides have been significantly associated with an increased risk of developing depressive disorders [3]. Studies consistently demonstrate that individuals aged 40 and older with depression have a 1.55-fold higher likelihood of experiencing comorbidities compared to their healthy counterparts. Among middle-aged and elderly populations [4], there is also a heightened susceptibility to metabolic disorders. These findings underscore the importance of addressing the comorbidity between depression and MetS in these vulnerable groups. Identifying metabolic biomarkers associated with depressive symptoms may offer valuable insights into the diagnosis and prognosis of depression. Insulin resistance (IR), defined as a reduced sensitivity of the body to insulin’s physiological effects, impairs normal biological processes [5]. It is the primary pathophysiological mechanism underlying MetS and type 2 diabetes. Previous research has shown that insulin levels and the IR index increase during active depressive episodes but remain unchanged during remission [6]. Interestingly, while the prevalence of depression among patients with type 1 diabetes is comparable to that of the general population, individuals with type 2 diabetes exhibit approximately double the average rate of depression [7]. This increased prevalence of depression in type 2 diabetes has been positively correlated with IR [8]. Currently, there is no universally accepted, highly practical method for measuring IR in clinical settings [9, 10]. While gold-standard techniques, such as the euglycemic insulin clamp and intravenous glucose tolerance testing, are used in academic research, their invasive nature and high cost render them impractical for widespread clinical use. The homeostasis model assessment-estimated IR (HOMA-IR) index, widely adopted for assessing IR, also has notable limitations. It primarily evaluates β-cell function alongside IR, making it less effective for individuals undergoing insulin therapy or those without functional beta cells [9]. To overcome these limitations, the triglyceride-glucose (TyG) index has been proposed. It has demonstrated greater efficacy than HOMA-IR in assessing IR in both diabetic and non-diabetic populations [11]. The TyG index has already been extensively used as a predictive and diagnostic biomarker for cardiovascular disease (CVD) and metabolic-related disorders [12]. Recent studies have also identified a link between TyG levels and the onset of depression. Depressed individuals exhibit elevated TyG indices compared to the general population [13], and depression severity has been positively correlated with TyG levels [14]. However, it is important to note that existing research findings are derived solely from cross-sectional observational studies [15].

**Figure 1.**
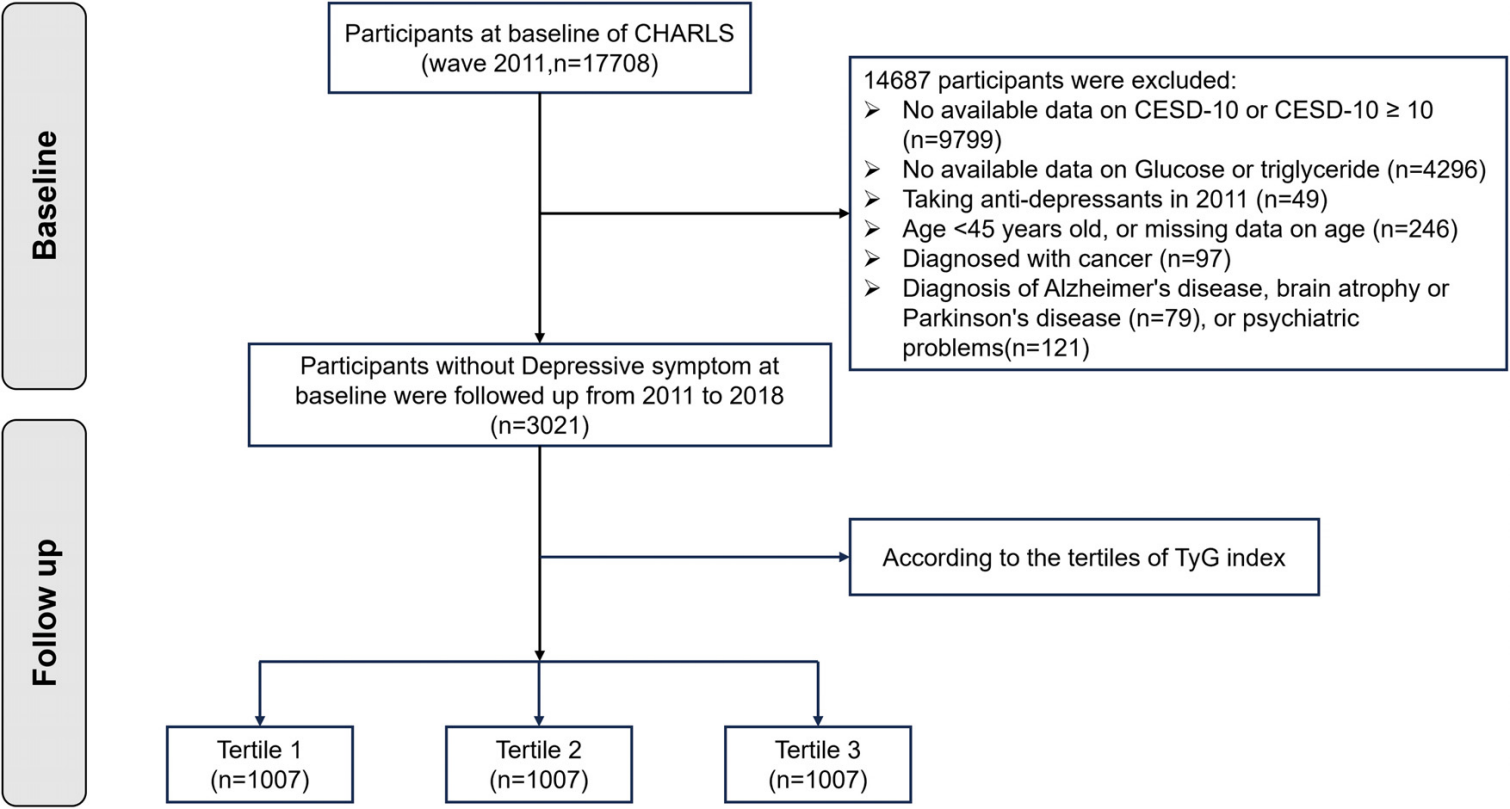
**Flowchart of study participants.** CHARLS: China Health and Retirement Longitudinal Study; TyG: Triglyceride-glucose.

Addressing these knowledge gaps, we extracted data from the China Health and Retirement Longitudinal Study (CHARLS) database to explore the relationship between the TyG index and depression, while also assessing its potential as a predictive indicator. This preliminary cohort research investigates the association between the TyG index and the risk of depression, emphasizing the significance of metabolic factors in mental health among middle-aged and elderly Chinese adults.

## Materials and methods

### Study design and participants

This observational study utilized data from the CHARLS, as previously described. To ensure a representative sample, a multi-phase probability sampling method was employed during the baseline survey. To date, five follow-up surveys have been conducted—in 2011, 2013, 2015, 2018, and 2020. During each wave, trained interviewers gathered sociodemographic and health-related data through face-to-face interviews. Additionally, health professionals performed medical examinations and collected medical information and blood samples during four of these waves (2011, 2013, 2015, and 2018). In the 2011 wave, 17,708 participants were initially screened, with 3021 selected and categorized into three subgroups based on baseline TyG tertiles. The remaining 14,687 participants were excluded due to the following reasons: missing data on CESD-10 or a CESD-10 score ≥10 (*n* ═ 9799), missing data on glucose or triglyceride (*n* ═ 4296), age <45 years or missing age data (*n* ═ 246), taking antidepressants at baseline (*n* ═ 49), a cancer diagnosis (*n* ═ 97), a diagnosis of Alzheimer’s disease, brain atrophy, or Parkinson’s disease (*n* ═ 79), or other psychiatric problems (*n* ═ 121). The selection criteria are detailed in Figure 1. This research framework adheres to the Strengthening the Reporting of Observational Studies in Epidemiology (STROBE) guidelines [16].

### Assessment of depression

This research employed the 10-item Center for Epidemiologic Studies Depression Scale (CES-D-10) [17] to measure baseline depression levels. Scores on the CES-D-10 ranged from 0 to 30, with higher scores indicating more severe depressive symptoms. Participants scoring 10 or above were classified as experiencing elevated depression. The cut-off point was validated as effective for identifying depressive status among elderly individuals in China [18]. The CES-D-10 scale used a 4-point Likert system to assess the frequency of depressive symptoms over the past week, ranging from “rarely (less than 1 day, 0 points)” to “all of the time (5–7 days, 3 points).” Notably, two items related to positive affect (i.e., feeling happy and feeling hopeful) were scored inversely [18].

### Assessment of TyG index

Blood samples were collected by trained personnel following clinical standard operating procedures. Both fasting blood glucose (FBG) and serum lipid parameters were measured using an enzyme colorimetric assay. Additionally, the TyG index was calculated using the formula: TyG index ═ ln (fasting TG [mg/dL] × FPG [mg/dL]/2).

### Covariates

At baseline (2011), trained interviewers collected data on sociodemographic and health-related characteristics using a standardized questionnaire. Sociodemographic data included age, gender (male or female), residence (rural or urban), marital status (married or other), and education level (no formal education, primary school, or high school and above). Health-related status contained alcohol consumption (never, former, current), smoking status (never, former, current), sleep time (<6, >═6), nap time and physician-diagnosed medical conditions (diabetes, hypertension, heart diseases, kidney disease, arthritis, digestive disease, stroke and dyslipidemia). Blood pressure (BP) was measured three times at 45-s intervals using a digital sphygmomanometer, following at least 5 min of seated rest. Body weight and height were measured using a digital scale and stadiometer, with a precision of 0.1 kg and 0.1 cm, respectively. Body mass index (BMI) was calculated using the formula BMI ═ body weight (kg)/height (m^2^). Additionally, blood samples were analyzed for various biomarkers, including hemoglobin, blood urea nitrogen (BUN), high-density lipoprotein cholesterol (HDL-C), low-density lipoprotein cholesterol (LDL-C), uric acid (UA), serum creatinine, C-reactive protein (CRP), white blood cell count (WBC), and glycosylated hemoglobin A1c (HbA1c), following standardized laboratory protocols.

### Outcome and follow-up

The outcome of the current research was depression, measured using the self-reported CES-D-10 scale, as described in previous studies. Participants were monitored from the initial assessment in 2011 until they either showed symptoms of depression or completed the final survey in 2018, whichever came first.

### Ethical statement

The CHARLS research project was granted ethical approval by Peking University’s Institutional Review Board, holding approval codes IRB00001052-11015 for household surveys and IRB00001052-11014 for blood sample collection. All participants gave informed consent in writing before taking part in the study.

### Statistical analysis

The means and standard deviations (SDs) or medians (IQRs) were reported for continuous variables, while categorical variables were described using counts and percentages. Differences among datasets—categorized as following a normal distribution, a skewed distribution, or representing categorical variables—were assessed using one-way analysis of variance (ANOVA), Kruskal–Wallis tests, and chi-square tests, respectively. The mice R package was employed for multiple imputation to address missing values and complete the dataset. Cox regression models were used to estimate the hazard ratio (HR) and 95% confidence interval (CI) for the association between TyG and depression. The proportional hazards assumption of the Cox models was evaluated using the Schoenfeld residual test [19], which indicated no significant violations by the exposure or any covariates. Additionally, Kaplan–Meier curves and log-rank tests were used to assess the cumulative hazard of depression over time. A linearity test was performed using the continuous variable represented by the median value for each group. Four fundamental models were developed based on both univariate and multivariate cox analyses (Supplementary Table 2). Model 1 served as the basic (unadjusted) model. Model 2 accounted for factors such as gender, residence, education level, smoking status, nighttime activity, and waist circumference. Model 3 included all variables from Model 2, along with serum creatinine, CRP, and BUN. Model 4 incorporated all variables in Model 3, in addition to the presence of arthritis and kidney disease. Across all four models, the baseline group was defined as the lowest tertile of the TyG index. Moreover, a restricted cubic splines (RCS) model was employed to examine the potential dose-response relationship between the TyG index and the likelihood of developing depression, adjusting for the aforementioned covariates.

**Table 1:** Baseline characteristics of participants according to the TyG index tertiles

**Characteristics**	**All (*n* ═ 3021)**	**TyG index**			***P* value**
		**Tertile 1 (*n* ═ 1007)**	**Tertile 2 (*n* ═ 1007)**	**Tertile 3 (*n* ═ 1007)**	
Age, mean (SD)	56.9 (8.38)	56.5 (8.55)	57.2 (8.60)	57.0 (7.97)	0.152
Gender, *n* (%)					
Male	1477 (48.9)	573 (56.9)	478 (47.5)	426 (42.3)	
Female	1544 (51.1)	434 (43.1)	529 (52.5)	581 (57.7)	
Residence, *n* (%)					0.001
Rural	2436 (80.6%)	848 (84.2)	808 (80.2)	780 (77.5)	
Urban	585 (19.4%)	159 (15.8)	199 (19.8)	227 (22.5)	
Education, *n* (%)					0.612
No formal education	1140 (37.7)	379 (37.6)	390 (38.7)	371 (36.8)	
Primary school	1465 (48.5)	493 (49.0)	469 (46.6)	503 (50.0)	
High school or above	416 (13.8)	135 (13.4)	148 (14.7)	133 (13.2)	
Marital status, *n* (%)					0.418
Married	2813 (93.1)	942 (93.5)	929 (92.3)	942 (93.5)	
Others	208 (6.89)	65 (6.45)	78 (7.75)	65 (6.45)	
SBP^b^(mmHg), mean (SD)	128 (19.5)	124 (18.3)	128 (20.2)	131 (19.5)	<0.001
DBP^b^ (mmHg), mean (SD)	74.9 (11.2)	73.2 (10.6)	74.7 (11.3)	76.6 (11.3)	<0.001
BMI^b^ (kg/m^2^), mean (SD)	24.0 (3.80)	23.0 (3.50)	24.0 (3.83)	25.1 (3.75)	<0.001
Waist circumference^b^ (cm), median (IQR)	84.2 [77.8;91.0]	81.4 [75.8;87.5]	84.5 [78.0;91.0]	88.0 [80.0;94.5]	<0.001
Alcohol consumption, *n* (%)					0.041
Never	1769 (58.6)	556 (55.2)	595 (59.1)	618 (61.4)	
Former	207 (6.85)	66 (6.55)	72 (7.15)	69 (6.85)	
Current	1045 (34.6)	385 (38.2)	340 (33.8)	320 (31.8)	
Smoking status, *n* (%)					
Never	1853 (61.3)	570 (56.6)	629 (62.5)	654 (64.9)	<0.001
Former	239 (7.91)	80 (7.94)	73 (7.25)	86 (8.54)	
Current	929 (30.8)	357 (35.5)	305 (30.3)	267 (26.5)	
Sleep time^b^ (h), *n* (%)					0.305
<6	1288 (42.6)	418 (41.5)	449 (44.6)	421 (41.8)	
>═6	1733 (57.4)	589 (58.5)	558 (55.4)	586 (58.2)	
Nap time^b^ (h), median (IQR)	2.00 [0.00;60.0]	2.00 [0.00;60.0]	2.00 [0.00;60.0]	3.00 [0.00;60.0]	0.184
Hemoglobin^b^ (g/dL), mean (SD)	14.4 (1.92)	14.3 (1.91)	14.3 (1.93)	14.5 (1.92)	0.011
HbA1c, mean (SD)	5.10 [4.90;5.40]	5.10 [4.80;5.30]	5.10 [4.80;5.30]	5.20 [4.90;5.50]	<0.001
HDL (mg/dL), mean (SD)	51.2 (13.5)	57.7 (13.0)	52.2 (12.3)	43.6 (11.2)	<0.001
LDL (mg/dL), mean (SD)	115 (31.0)	108 (28.6)	120 (29.7)	118 (33.3)	<0.001
BUN^b^ (mg/dL), mean (SD)	15.4 (4.07)	15.9 (4.27)	15.2 (3.98)	14.9 (3.90)	<0.001
UA^b^ (mg/dL), mean (SD)	4.34 (1.12)	4.20 (1.09)	4.29 (1.07)	4.55 (1.17)	<0.001
Serum creatinine (mg/dL), mean (SD)	0.77 (0.16)	0.76 (0.16)	0.76 (0.16)	0.78 (0.16)	0.025
CRP (mg/L), mean (SD)	0.92 [0.51;1.81]	0.75 [0.44;1.57]	0.87 [0.50;1.73]	1.15 [0.65;2.19]	<0.001
WBC^b^ (thousands/UL), mean (SD)	6.05 (1.61)	5.88 (1.56)	5.98 (1.61)	6.30 (1.63)	<0.001
Depressive symptom *n* (%)	1782 (59.0)	539 (53.5)	605 (60.1)	638 (63.4)	<0.001
Hypertension, *n* (%)	629 (20.8)	140 (13.9)	192 (19.1)	297 (29.5)	<0.001
Kidney disease, *n* (%)	165 (5.46)	58 (5.76)	60 (5.96)	47 (4.67)	0.390
Dyslipidemia, *n* (%)	249 (8.24)	56 (5.56)	69 (6.85)	124 (12.3)	<0.001
Arthritis, *n* (%)	837 (27.7)	231 (22.9)	301 (29.9)	305 (30.3)	<0.001
Digestive disease, *n* (%)	596 (19.7)	201 (20.0)	204 (20.3)	191 (19.0)	0.748
Heart disease, *n* (%)	267 (8.84)	73 (7.25)	72 (7.15)	122 (12.1)	<0.001
Diabetes, *n* (%)	119 (3.94)	21 (2.09)	29 (2.88)	69 (6.85)	<0.001
Stroke, *n* (%)	37 (1.22)	15 (1.49)	12 (1.19)	10 (0.99)	0.595

^a^*P* value was calculated by ANOVA test for continuous variables and chi-square test for categorical variables; ^b^Data for some participants were missing. SBP: Systolic blood pressure; DBP: Diastolic blood pressure; BMI: Body mass index; HbA1c: Glycosylated hemoglobin A1c; HDL: High density lipoprotein; LDL: Low density lipoprotein; BUN: Blood urea nitrogen; UA: Uric acid.

**Table 2:** Hazard ratios of triglyceride-glucose index for depression in the study participants

**TyG index**	**HR (95% CI)**							
	**Model 1**	***P* value**	**Model 2**	***P* value**	**Model 3**	***P* value**	**Model 4**	***P* value**
TyG index*	1.39 (1.28–1.52)	<0.001	1.39 (1.27–1.52)	<0.001	1.35 (1.24–1.48)	<0.001	1.34 (1.23–1.47)	<0.001
*Tri-sectional TyG*								
Tertile 1	Reference		Reference		Reference		Reference	
Tertile 2	1.17 (0.86–1.15)	0.009	1.12 (0.99–1.26)	0.055	1.10 (0.98–1.24)	0.099	1.08 (0.96–1.22)	0.183
Tertile 3	1.28 (0.78–1.15)	<0.001	1.25 (1.12–1.41)	<0.001	1.22 (1.07–1.37)	0.001	1.20 (1.06–1.35)	0.003
*P* for trend	<0.001		<0.001		0.001		0.002	

a. Unadjusted for covariates; b. Adjusted for Gender, Residence, Education, Smoking status, Night time, Waist circumstance, BMI; c. Adjusted for Gender, Residence, Education, Smoking status, Night time, Waist circumstance, BMI, Serum creatinine, CRP, BUN; d. Adjusted for Gender, Residence, Education, Smoking status, Night time, Waist circumstance, BMI, Serum creatinine, CRP, BUN, Arthritis, Kidney disease. *TyG index as a continuous variable. HR: Hazard ratio; CI: Confidence interval; TyG: Triglyceride-glucose.

**Figure 2.**
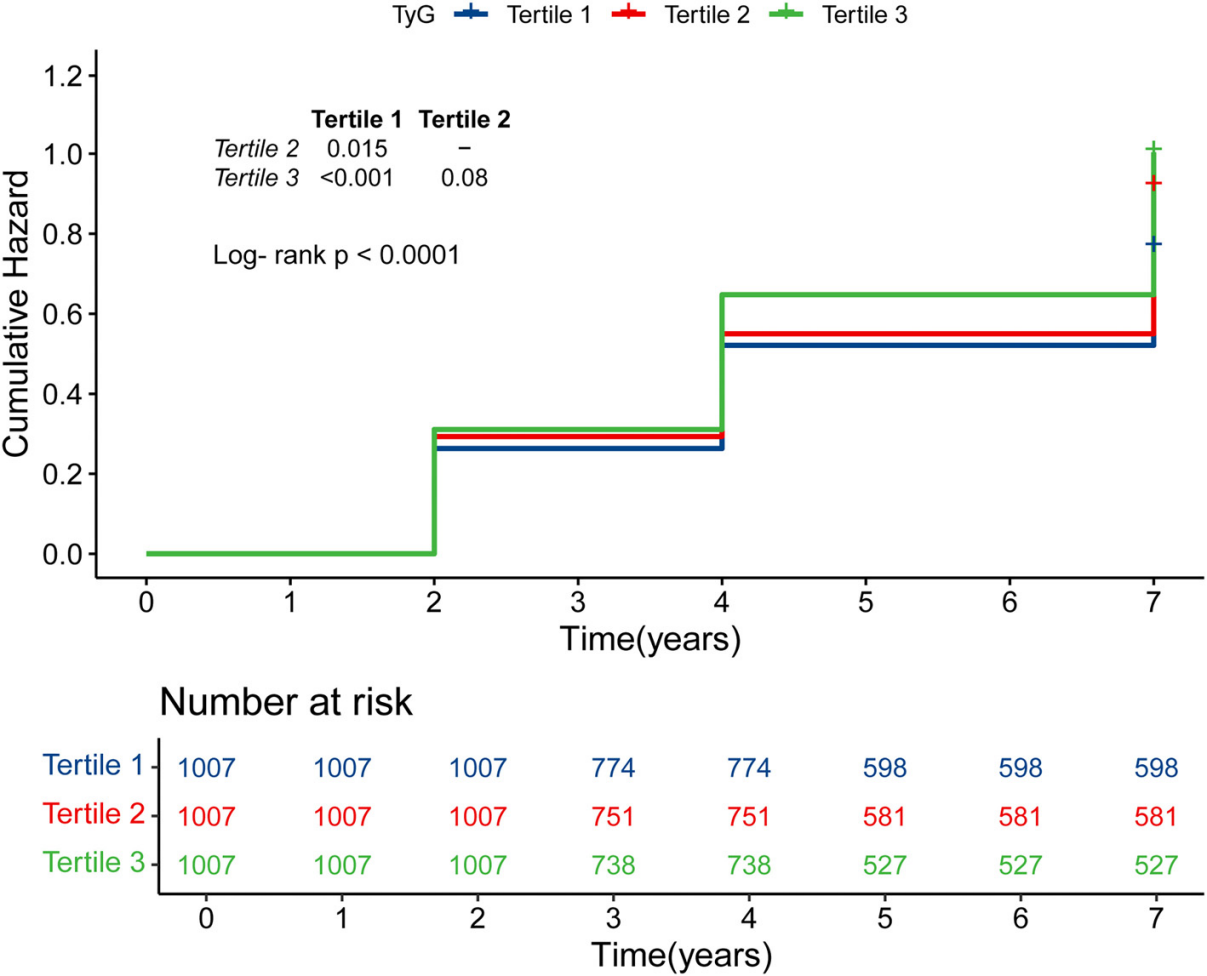
**Kaplan–Meier curves for the cumulative incidence of depressive symptoms.** Tertile 1 is TyG ≤8.28; Tertile 2 is TyG >8.28 but ≤8.77; Tertile 3 is TyG >8.77. TyG: Triglyceride-glucose.

Subgroup analysis assessed the TyG index’s prognostic consistency across various subgroups. These subgroups were defined based on gender (male, female), marital status (married, other), residence (urban, rural), education level (no formal education, primary school, or high school and above), smoking status (never, former, or current), drinking status (never, former, or current), sleep time (<6, ≥6) and the presence of depression-related internal medicine diseases, such as hypertension, diabetes, dyslipidemia, kidney disease, and heart disease. The statistical analyses were conducted using RStudio 4.2.1. All statistical tests were two-tailed, and significance was determined at *P* < 0.05.

## Results

### Baseline characteristics

The baseline characteristics of the study population were presented both overall and in quartiles of TyG (Table 1). The current cohort analysis included a total of 3021 participants, with a mean age of 56.9 ± 8.38 years. Participants in T2-3 of TyG were characterized by advanced age and a higher proportion of females, along with higher SBP, DBP, BMI, waist circumference, hemoglobin, HbA1c, LDL, UA, serum creatinine, CRP, and WBC levels. Additionally, there was a notable increase in the percentage of individuals who had never smoked or consumed alcohol, alongside a higher incidence of conditions, such as hypertension, dyslipidemia, arthritis, heart disease, and diabetes (all *P* < 0.05). In contrast HDL and BUN levels in the T2-3 group of TyG were significantly lower than those in T1 group (all *P* < 0.001). Furthermore, no statistical differences were found in education level, marital status, sleep duration, nap time, and the prevalence of kidney disease, digestive disorders, and stroke (all *P* > 0.05).

### Association between TyG and depression

**Figure 3.**
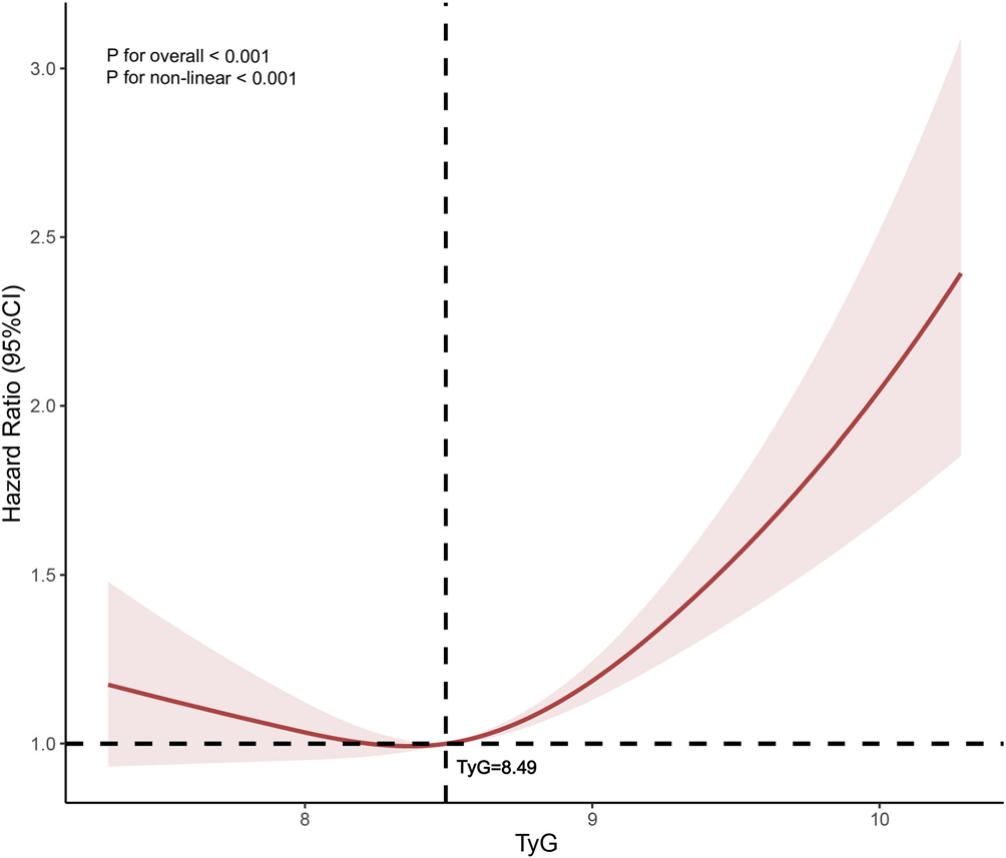
**The adjusted restricted cubic spline curves depict the relationship between depressive symptoms and the TyG index, accounting for various covariates.** The bold central line illustrates the estimated adjusted hazard ratio, while the shaded bands around it mark the 95% confidence interval. A vertical dashed line highlights the TyG threshold value of 8.49, and a horizontal dashed line indicates the baseline hazard ratio of 1.0. The analysis incorporates adjustments for factors, such as gender, residence, education level, smoking status, nighttime habits, waist circumference, BMI, serum creatinine, CRP levels, BUN, arthritis, and kidney disease. TyG: Triglyceride-glucose; BMI: Body mass index; CRP: C-reactive protein; BUN: Blood urea nitrogen; CI: Confidence interval.

During a median follow-up period of 7 years, depression was identified in 1782 participants (58.9%). The incidence rates of depression across tertiles (T1–T3) of the TyG index were 30.2%, 34.0%, and 35.8%, respectively (Supplementary Table 1). Figure 2 illustrates a steady increase in the cumulative hazard of depression from T1–T3, with a statistically significant difference (log-rank test, *P* < 0.001). Table 2 shows that for every 1.0-unit increase in the TyG index, the HR for depression was 1.39 (95% CI: 1.28–1.52) in the unadjusted Model 1. Although the strength of this association slightly diminished, it remained significant in the partially adjusted Model 2 (HR: 1.39, 95% CI: 1.27–1.52), the fully adjusted Model 3 (HR: 1.35, 95% CI: 1.24–1.48), and the comprehensive Model 4 (HR: 1.34, 95% CI: 1.23–1.47). Consistent with these findings, Model 4 also revealed that the fully adjusted HRs (95% CIs) for participants in T2 and T3 of the TyG index were 1.08 (0.96–1.22) and 1.20 (1.06–1.35), respectively, compared to those in T1.Furthermore, a multivariable-adjusted RCS analysis identified a significant “J”-shaped dose-response relationship between the TyG index and the risk of depression (*P* for overall trend < 0.001; *P* for nonlinear trend < 0.001). A TyG index value of 8.49 was identified as the baseline risk point, where the HR equals 1.0. Below this threshold, the HR remained relatively stable and close to 1.0, suggesting that lower TyG levels have minimal impact on depression risk. However, TyG values exceeding 8.49 were associated with a marked increase in the HR, reflecting a significantly heightened risk of depression (Figure 3). Additionally, ROC curve analysis was performed to evaluate the predictive value of depression indices after adjusting for covariates. A statistically significant difference in the AUC was observed between models with and without the inclusion of the TyG index (AUC-a: 0.651 vs AUC-b: 0.662, *P* ═ 0.009). This finding highlights the TyG index’s predictive ability for depression (Supplementary Figure 1).

### Subgroup analysis

**Figure 4.**
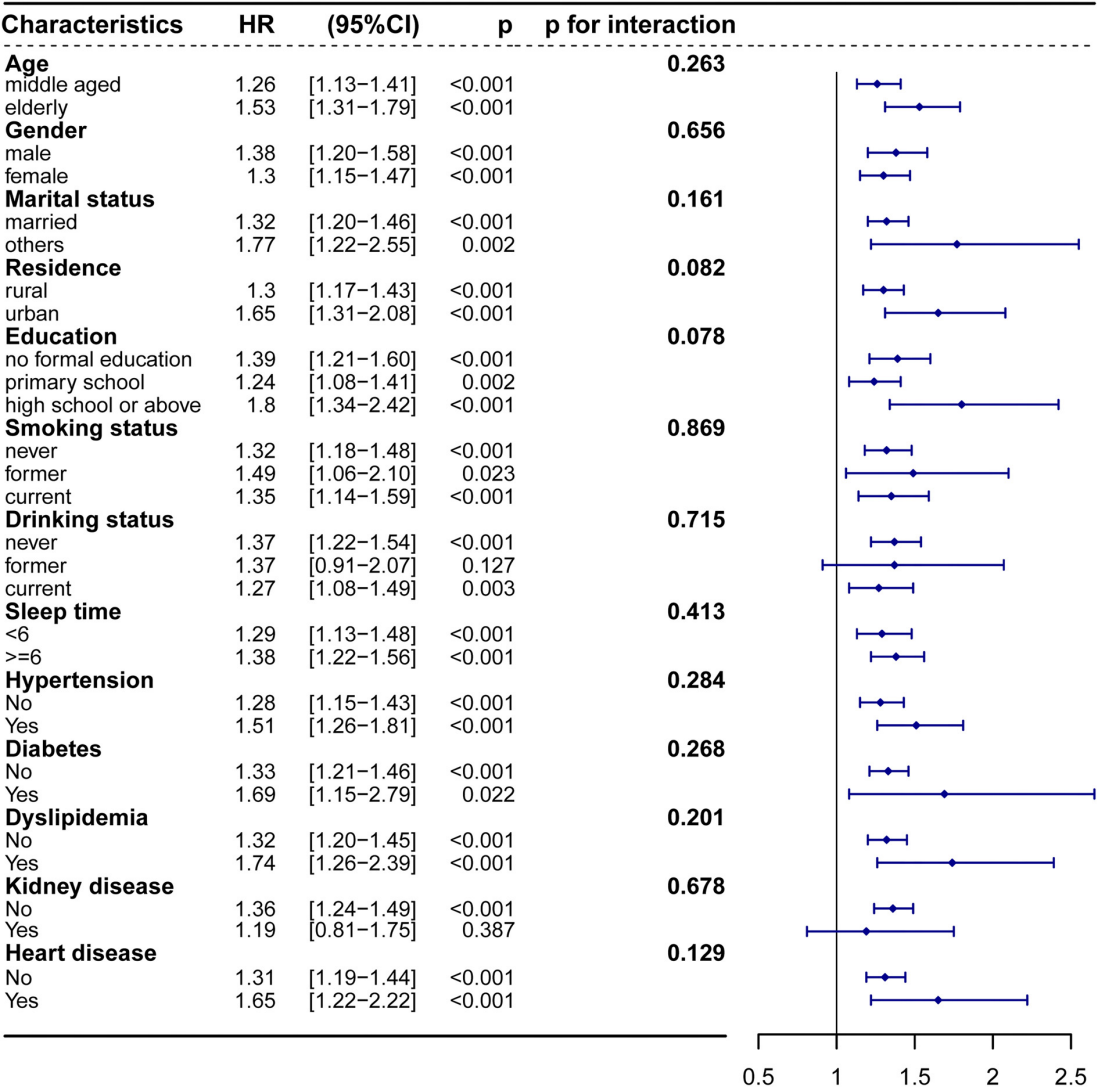
**Subgroup and interaction analyses between the TyG (continual) and depressive symptoms across various subgroups.** TyG: Triglyceride-glucose; CI: Confidence interval; HR: Hazard ratio.

The association between TyG and depression was further evaluated across various subgroups. After adjusting for potential confounders, our analysis did not find significant evidence of variation in the risk of depression based on CESD-10 scores (*P* for interaction > 0.05 for all) (Figure 4 and Supplementary Table 3).

## Discussion

In this large-scale, nationwide, longitudinal cohort study involving 3021 middle-aged and elderly individuals in China, a significant association was observed between elevated TyG levels and an increased risk of new-onset depression, even after adjusting for potential confounding factors. Subgroup analysis further validated the robustness of this relationship. Additionally, a notable dose-response relationship between TyG levels and the incidence of depression was identified using RCS analysis. These findings suggest that TyG could serve as a valuable biomarker for predicting the risk of developing depressive disorders. Regular monitoring and management of TyG levels may, therefore, play a role in preventing depression. MetS has emerged as a critical global public health concern, characterized by IR, abdominal obesity, dyslipidemia, chronic inflammation, type 2 diabetes mellitus (T2DM), and an increased risk of CVDs [20]. Over recent decades, the prevalence of MetS has surged, now affecting approximately 33.9% of the population—around 450 million individuals—in China alone [21–23]. The age group of 40–60 years is particularly vulnerable to developing MetS [24]. Furthermore, mounting evidence strongly supports a robust association between MetS, IR/T2DM, and an increased susceptibility to depressive disorders.

IR, a core pathophysiological mechanism of MetS, has been linked to depressive disorders. Nationwide studies have reported an association between higher IR markers, such as HOMA-IR scores in Korea [25] and the TyG index in the USA [26], with an increased prevalence of depressive moods. Due to its non-invasive and straightforward measurement, the TyG index holds significant potential for clinical use in evaluating IR. Previous research highlights the relationship between the TyG index and depression. For example, a cross-sectional study found that patients with major depressive disorder (MDD) exhibited significantly higher TyG levels compared to non-MDD individuals (8.77 [8.34–9.17] vs 8.62 [8.18–9.01], *P* < 0.001) [27]. Similarly, a nationwide study observed that individuals with suicidal ideation had significantly higher TyG indices compared to those without (8.71 ± 0.68 vs 8.60 ± 0.66, *P* < 0.001) [28]. Furthermore, TyG-related indices—such as TyG-BMI, TyG-WC, and TyG-WHtR—also demonstrate a strong positive correlation with depressive symptoms, particularly in premenopausal women [29]. Our results further demonstrate that compared to T1, the HR for T2 is 1.08 (0.96–1.22, *P* ═ 0.183), while the HR for T3 compared to T1 is 1.20 (1.06–1.35, *P* ═ 0.003) after adjusting for all covariates (Table 2). In the Kaplan–Meier curve analysis, a significant difference in cumulative HR was observed between T1 (TyG ≤8.28) and T2 (8.28 < TyG ≤ 8.77) (*P* ═ 0.015), as well as between T1 and T3 (TyG > 8.77) (*P* < 0.001). This indicates that high levels of TyG are not only closely related to depression, but also increase the risk of developing these disorders. Interestingly, while the risk of depression initially decreased with higher TyG levels, it subsequently increased as TyG above 8.49. However, the HR for TyG above 8.49 remained lower compared to levels above 8.49, with a CI less than 1 (Figure 3). Based on the cox regression model, we propose that the risk of depression rises significantly when TyG levels reach or exceed 8.49. Nevertheless, the optimal TyG cutoff for predicting depression may vary across age groups. In a cross-sectional study of 8970 individuals with prediabetes and diabetes, a positive correlation was observed between the TyG index and depression prevalence. Interestingly, the relationship differed across ethnic groups, with a U-shaped association in Hispanic populations and a turning point at a TyG index of 8.85 [30]. Despite these findings, it remains unclear whether low TyG levels may increase susceptibility to depression below a certain threshold. Given the potential impact of malnutrition—especially in middle-aged and elderly individuals [31, 32] or those with comorbidities [33]—further epidemiological studies are warranted to explore depression prevalence among populations with low TyG levels. Such research could help clarify the complex relationship between TyG and depression.

Subgroup analysis revealed a notable gender disparity in depression prevalence. Consistent with prior studies, women were approximately twice as likely to develop depression compared to men [34]. However, within the T2 and T3 groups, male participants exhibited a higher risk of depression than their female counterparts (Supplementary Table 3). We hypothesize that this discrepancy may stem from gender differences in the prevalence of MetS [35]. Married individuals showed a significantly lower risk of depressive disorders compared to those who were divorced or unmarried. This finding aligns with research highlighting the protective effects of intimate relationships and marital status on mental health, likely due to the stress-buffering benefits of social support [36, 37]. Despite the skewed rural–urban participant ratio (80.6% rural, 19.4% urban), urban residents demonstrated a higher susceptibility to depression. Similar patterns have been observed in a U.S. cross-sectional study, which noted disparities in mental illness prevalence between urban and rural populations. These differences could reflect the benefits of improved urban living conditions on mental well-being or, conversely, the increased adverse life events faced by urban dwellers [38]. These findings suggest urbanization should be considered in mental health resource allocation. Consistent with prior studies [39–41], our findings further underscore the heightened risk of depression among individuals with internal medicine conditions, such as hypertension, diabetes, dyslipidemia, kidney disease, and heart disease, particularly diabetes and dyslipidemia. As both conditions are established risk factors for MetS, this strengthens the link between MetS and depression. This study supports the association between the TyG index (an indicator of MetS) and depression in middle-aged and elderly populations, consistent with previous research [42]. Our prospective cohort study suggests that an elevated TyG index may be a potential risk factor for depression. However, the relationship may be bidirectional—depression can contribute to MetS development, and MetS may increase depression risk. Poor lifestyle choices and negative life events, such as alcohol abuse, sleep disorders, lack of physical exercise, and unhealthy diets, are shared risk factors for both MetS and mood disorders [43, 44]. Lifestyle modifications can significantly alleviate depressive symptoms and reduce MetS incidence [45]. Chronic low-grade inflammation is a shared mechanism underlying both MetS and depression. Coexistence of these conditions disrupts the hypothalamic-pituitary-adrenal (HPA) axis, triggering inflammatory responses [46]. HPA axis overactivation contributes to visceral fat accumulation, hypercortisolemia, fatty acid release, very-LDL (VLDL) production, and hyperglycemia [47]. Furthermore, metabolic-induced inflammation promotes cytokine release, with peripheral cytokines crossing the blood–brain barrier to activate microglia and other immune cells, disrupting neurotransmitter function [48]. Recent studies have identified genetic variants linked to both mood disorders and cardiometabolic conditions, including *CACNA1D, FTO, BDNF, POMC, IGF* [49]. Shared genetic pathways involve corticotropin-releasing hormone signaling, serotonin and dopamine receptor signaling, circadian rhythm regulation, and leptin signaling. Genetic risk scores associated with obesity and inflammation correlate strongly with MDD, particularly neurovegetative symptoms [50]. Similarly, genes linked to obesity, leptin, MTHFR, and serotonin receptor 2C are implicated in both MetS and schizophrenia [51]. These findings suggest a shared genetic vulnerability contributing to the comorbidity of MetS and psychiatric disorders.

However, several limitations must be acknowledged. Firstly, depression was assessed using a self-reported scale, which may reflect symptoms of depression rather than a clinical diagnosis of depressive disorder. Secondly, while multivariate Cox models were employed to adjust for potential confounders, residual confounding cannot be entirely ruled out. For instance, age distribution was not included in the multivariate regression model, highlighting the need for future research to explore the relationship between the TyG index and depression across all age groups. Thirdly, participants with a low TyG index at baseline may have experienced changes in their index over time due to factors, such as lifestyle modifications, aging, or the progression of metabolic conditions. By relying solely on baseline measurements, this study may not fully account for the dynamic nature of the TyG index and its evolving association with depressive symptoms. Lastly, since the study only included individuals aged 45 years or older, caution should be exercised when generalizing these findings to younger populations or the broader public.

## Conclusion

The extensive, nationally representative sample in this study enabled us to investigate the association between TyG and the risk of depression. Our findings offer new evidence to support primary prevention strategies aimed at depression, highlighting potential opportunities to reduce its incidence through early recognition and intervention.

## Supplemental data

Supplemental data are available at the following link: https://www.bjbms.org/ojs/index.php/bjbms/article/view/11800/3709.

## Data Availability

The datasets generated and analyzed during the current study are available in the CHARLS website (https://charls.charlsdata.com/).
